# Robust Co alloy design for Co interconnects using a self-forming barrier layer

**DOI:** 10.1038/s41598-022-16288-y

**Published:** 2022-07-19

**Authors:** Cheol Kim, Geosan Kang, Youngran Jung, Ji-Yong Kim, Gi-Baek Lee, Deokgi Hong, Yoongu Lee, Soon-Gyu Hwang, In-Ho Jung, Young-Chang Joo

**Affiliations:** 1 Department of Materials Science & Engineering, Seoul National University, Seoul, 08826 Republic of Korea; 2 Research Institute of Advanced Materials (RIAM), Seoul National University, Seoul, 08826 Republic of Korea

**Keywords:** Engineering, Materials science, Nanoscience and technology

## Abstract

With recent rapid increases in Cu resistivity, *RC* delay has become an important issue again. Co, which has a low electron mean free path, is being studied as beyond Cu metal and is expected to minimize this increase in resistivity. However, extrinsic time-dependent dielectric breakdown has been reported for Co interconnects. Therefore, it is necessary to apply a diffusion barrier, such as the Ta/TaN system, to increase interconnect lifetimes. In addition, an ultrathin diffusion barrier should be formed to occupy as little area as possible. This study provides a thermodynamic design for a self-forming barrier that provides reliability with Co interconnects. Since Cr, Mn, Sn, and Zn dopants exhibited surface diffusion or interfacial stable phases, the model constituted an effective alloy design. In the Co-Cr alloy, Cr diffused into the dielectric interface and reacted with oxygen to provide a self-forming diffusion barrier comprising Cr_2_O_3_. In a breakdown voltage test, the Co-Cr alloy showed a breakdown voltage more than 200% higher than that of pure Co. The 1.2 nm ultrathin Cr_2_O_3_ self-forming barrier will replace the current bilayer barrier system and contribute greatly to lowering the *RC* delay. It will realize high-performance Co interconnects with robust reliability in the future.

## Introduction

Interconnection technology has been continuously developed to reduce resistance–capacitance (*RC*) delay and ensure reliability^1–3^. Recently, the resistivities of Cu interconnects have significantly increased due to the continuous scaling down of the interconnect pitch. To reduce the *RC* delay, beyond Cu (non-Cu) materials are attracting attention, and cobalt (Co) and platinum group metals (PGM) are the most studied^4–7^. The driving force for the development of beyond Cu interconnects is to lower the *RC* delay of the metal/barrier system and achieve robust interconnect reliability. Current Cu interconnects use a Ta/TaN diffusion barrier, so the resistivity of the metal/barrier system is substantially increased^8–10^. In a situation in which the M1 pitch narrows to 30 nm at the 3 nm node and the thickness of the barrier metal does not decrease to 2 nm or less, the proportion of pure metal cannot exceed 50%^11–13^. Therefore, to improve the performance of beyond Cu interconnects, an ultrathin (< 2 nm) diffusion barrier material should be developed to minimize the *RC* delay^14,15^. Despite the ultrathin width, robust diffusion barrier quality must be achieved. Since extrinsic failure due to metal ions has recently been reported for Co interconnects, the diffusion barrier must be sufficiently thin to block metal ion penetration^16,17^. Since a porous low-k dielectric is used, the quality of the diffusion barrier currently has a greater impact on interconnect reliability. This means that designs of diffusion barrier materials for metal/dielectric systems are very important, but there have been few studies on diffusion barriers suitable for Co/dielectric systems thus so far^18^.

The Cu-Mn alloy proposed by Koike improved the reliability of Cu interconnects by generating a MnSi_x_O_y_ self-forming barrier^19^. MnSi_x_O_y_ diffusion barriers were formed with thicknesses ranging from 2 to 8 nm, and they showed excellent diffusion barrier properties^20,21^. This study proposes for the first time a design for an ultrathin self-forming diffusion barrier suitable for the Co metal system and capable of ensuring robust reliability. It was noted that the self-forming barrier methodology is effective even for a node process with a narrower linewidth, and it is expected that extrinsic failures of Co interconnects can be controlled. Since the driving forces for self-formation of the barrier are diffusion and oxidation, defect-free interfacial adhesion properties can be obtained. Highly time-dependent dielectric breakdown (TDDB) resistance can be achieved through the formation of a high-density oxide diffusion barrier^22^. This also has the great advantage of low-k process compatibility because the barrier is formed by diffusion even in a nanoscale porous structure. A thermodynamic material is proposed for a Co-X alloy that generates a self-forming barrier and exhibits excellent diffusion barrier properties. By understanding the behavior of barrier self-formation and confirming interfacial diffusion and oxidation reactions for each alloy through thermodynamic calculations and experiments, it was possible to select self-formation barrier materials applicable to Co interconnects.

In this work, Co-Cr alloy showed the superior properties of self-forming barrier material by evaluating the quality of the diffusion barrier between five selected metals (Cr, Fe, Mn, Sn, Zn) through thermodynamic prediction for the interfacial stability phase. The formation of a Cr_2_O_3_ diffusion barrier at the dielectric interface resulted in very good barrier properties. Cr_2_O_3_ serves as a robust diffusion barrier in interconnects since it acts as a passivation layer with a low diffusion coefficient in structural materials such as stainless steel. Co-X alloy material design was shown to be a very effective design process because it matched well with the experimental results. The results of this study show that the Co/barrier/SiO_2_ system may be a promising solution for the Co interconnects.

## Results and discussion

**Figure 1:**
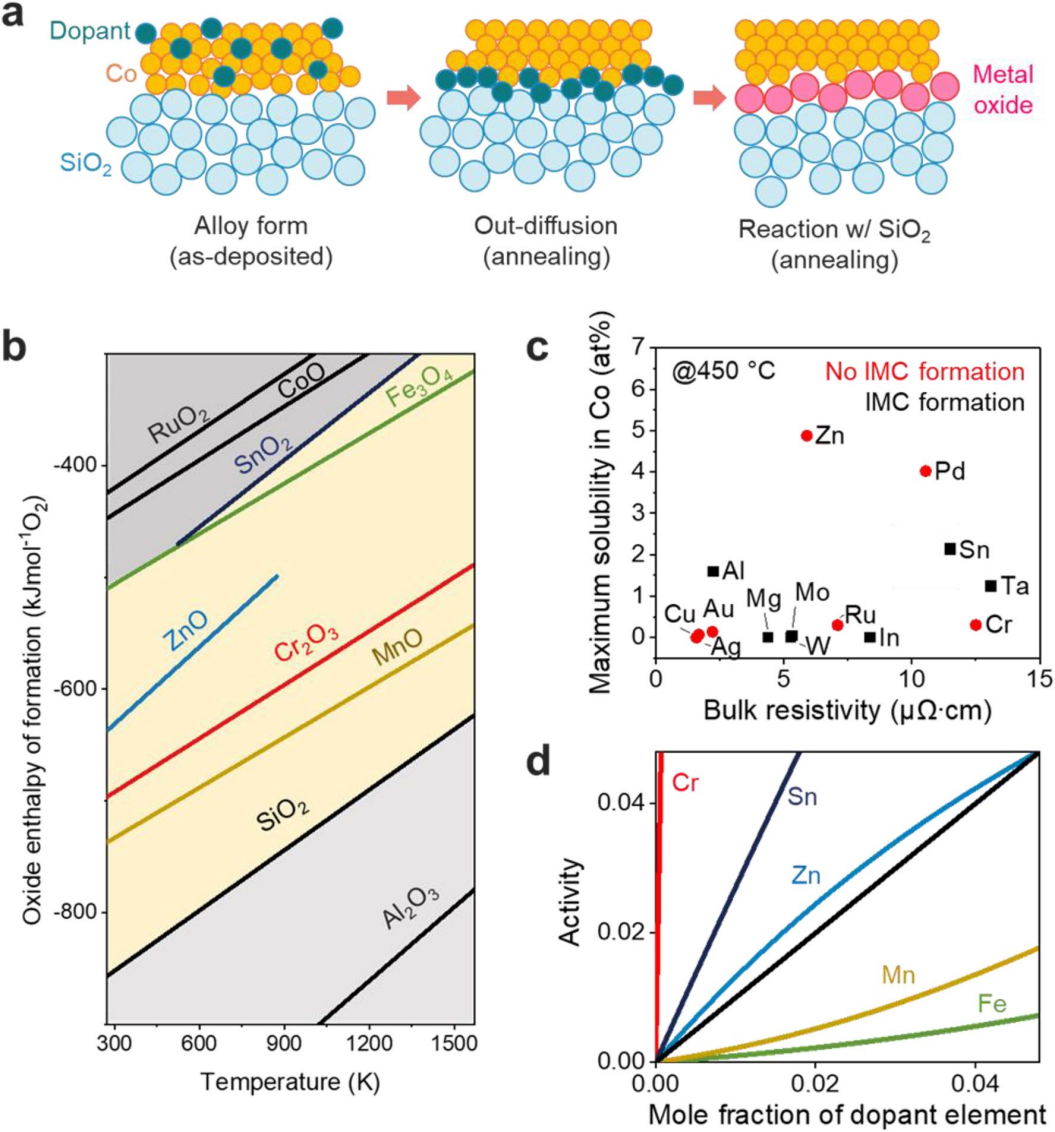
Cobalt alloy design for self-forming diffusion barrier in Co interconnects. (**a**) schematic illustration of the self-forming barrier formation mechanism during annealing process. (**b**) Ellingham diagram which provides Gibbs free energy of reactions between alloy elements and oxygen. (**c**) maximum solubility of alloy dopants in Co at 450 °C; the red dot is a Co alloy metal in which intermetallic compounds (IMC) do not exist, and when formed, it is indicated by a black box. (**d**) activity coefficient of each dopant in the Co at 450 °C using Factsage™ software.

Thermodynamic material design of a self-forming barrier for Co interconnects was performed by dividing the behavior of the self-forming barrier into three stages, as shown in Fig. 1a. For the self-forming reaction to occur, it is necessary to predict the extent of the oxidation reaction with the dielectric, so the oxidation tendency of the alloying element, Co, and SiO_2_ should be compared by using the Ellingham diagram. Since the alloying element does not exist only as a solid solution and must release, the solubility is low, and an intermetallic compound phase should not exist. The activity coefficient of the alloying element must be high for out-diffusion to occur. Thermodynamic design criteria necessary for the oxidation reaction and interfacial diffusion were established. Controlling surface diffusion by the dopant and reactions of the dopant with oxygen or silicon during the generation of the self-formation barrier layer is very important because this greatly influences barrier properties. First, to prevent extrinsic failure caused by metal ions, it is very important to know what phase is present at the SiO_2_ interface. Figure 1b contains an Ellingham diagram showing the standard free energy for oxidation of each element as a function of temperature. With the Ellingham diagram and thermodynamic calculations, the stable phase present at the interface can be predicted. When the dopant has an oxidation tendency between those of Co and Si, it is expected that a robust diffusion barrier with an appropriate thickness will be formed at the interface. This is because when the dopant has a lower oxidation tendency than Co, Co oxide is first formed because the driving force for the reaction of Co with oxygen is higher than that of the alloying element. When it has a higher tendency to form an oxide than Si, a diffusion barrier will be formed, but free Si may be present at the interface due to reactions with excess oxygen. Since free Si can also serve as an electrical path within the SiO_2_ dielectric, it will not be good in terms of electrical reliability. For example, in the case of a Cu-Mg alloy, desired behavior of the self-forming barrier behavior was confirmed, but it did not show robust barrier properties due to the high oxidation tendency of Mg^23^.

As described above, when materials can be screened by calculating the reactive phase at the Co-SiO_2_ interface, a self-forming barrier with excellent reliability can be designed for Co interconnects. The Ellingham diagram showed that a total of five metals (Cr, Fe, Mn, Sn, Zn) formed an excellent diffusion barrier. In Table 1, the thermodynamically stable phase of Co/SiO_2_ was calculated. It was predicted that Cr exists as Cr_2_O_3_ phase at the interface, Zn exists as Zn_2_SiO_4_, and Mn and Fe exist as a compound phase. Figure 1c illustrates the solubility limit of the Co-X alloy dopant at an annealing temperature (450 °C) and the presence or absence of intermetallic compound (IMC) formation. Since a solid solution is an unstable phase in the matrix, the driving force to escape to the interface is large. In general, dopants with low solubility were prioritized because alloys have large ranges of resistivity changes. When an IMC is formed, the driving force for out-diffusion is very low because the IMC is thermodynamically stable^24^. Cr is contained in Co alloys showing low solubility and does not form an IMC phase. In addition, Zn does not form IMC phases but has high solubility, Al element has low solubility and IMC phases are formed, and Sn element has high solubility and IMC phases are formed. Cr and Zn, which facilitate interfacial diffusion during annealing, are the most suitable alloying elements. Figure 1d shows the activity coefficients of the alloying elements selected with the design rule. The activity coefficient of a dopant in the Co alloy is another important indicator confirming out-diffusion behavior. A MnSi_x_O_y_ self-forming barrier formed by considering the activity coefficient in a Cu-Mn alloy has been reported for the first time^25^. The activity coefficient of each dopant in the Co alloy at 450 °C was calculated from its activity and mole fraction using Factsage™ software. In Co alloys, when the activity coefficient is higher than 1, the dopant is unstable in the Co matrix and tends toward out-diffusion. Conversely, when the activity coefficient is lower than 1, diffusion to the surface is difficult because the dopant is thermodynamically stable inside the Co matrix. Dopant metals showing activity coefficients greater than 1 include Cr and Sn, and they were expected to show out-diffusion behavior in Co alloys. In relation to thermodynamic designs of materials for self-forming Co barriers, calculation results for more alloying elements can be found in Tables S1–S3 and Fig. S1.

**Table 1:** Comparison of Co alloy elements for self-forming diffusion barrier (SFB): six design rules were used to determine which of the five dopants is suitable as a self-forming barrier material. The activity coefficient and solubility of the dopant are thermodynamically calculated values. The resistivity of each metal represents the bulk value of the resistivity.

Design rule of Co self-forming barrier	Cr	Zn	Mn	Fe	Sn
Oxide enthalpy of formation	Moderate	Moderate	Moderate	Moderate	Moderate
Reaction phase with Co/SiO_2_ at 450 °C	Cr_2_O_3_	Zn_2_SiO_4_	MnSiO_3_, CoSiO_3_	Fe–Co–Si compound	SnO_2_
Intermetallic compound (IMC) formation at 450 °C	Not formed	Not formed	Not formed	Not formed	Formed
Solubility at 450 °C (at%)	0.31	4.88	8.11	10.11	2.12
Activity coefficient of dopant	68.572	0.997	0.463	0.263	2.653
Resistivity (μΩ∙cm)	12.50	5.90	144	9.61	22.8

Table 1 shows the material design results for suitable alloying elements suitable for self-forming barriers in Co interconnects. From the thermodynamic design rule, it seems that Cr is most suitable for use as a self-forming barrier material. In the case of resistivity, it is necessary to check the resistivity of the Co-X alloy as a function of dopant concentration. In this study, the bulk resistivity of the material was considered, and when the resistivity was low, deterioration of the interconnect performance can be minimized. The electrical resistivity of these alloy films after the heat treatment can be found in Fig. S3. In the case of Cr, although the bulk resistivity was relatively high, interfacial diffusion during annealing was easy, and it is expected to form a Cr_2_O_3_ stable phase at the SiO_2_ interface. Cr_2_O_3_ is used as a passivation layer in structural materials such as stainless steel and is well known to slow the release of metal cations due to its high density and very low diffusion coefficient^26,27^. Cr_2_O_3_ passivation has not been in interconnection technology and seems to play a significant role in preventing extrinsic failure caused by metal ions. To evaluate the effectiveness of self-forming barriers in Co interconnects, comparative analyses of solubility and activity coefficients were conducted with Zn, Mn, Fe, and Sn in different intermetallic compounds.

**Figure 2:**
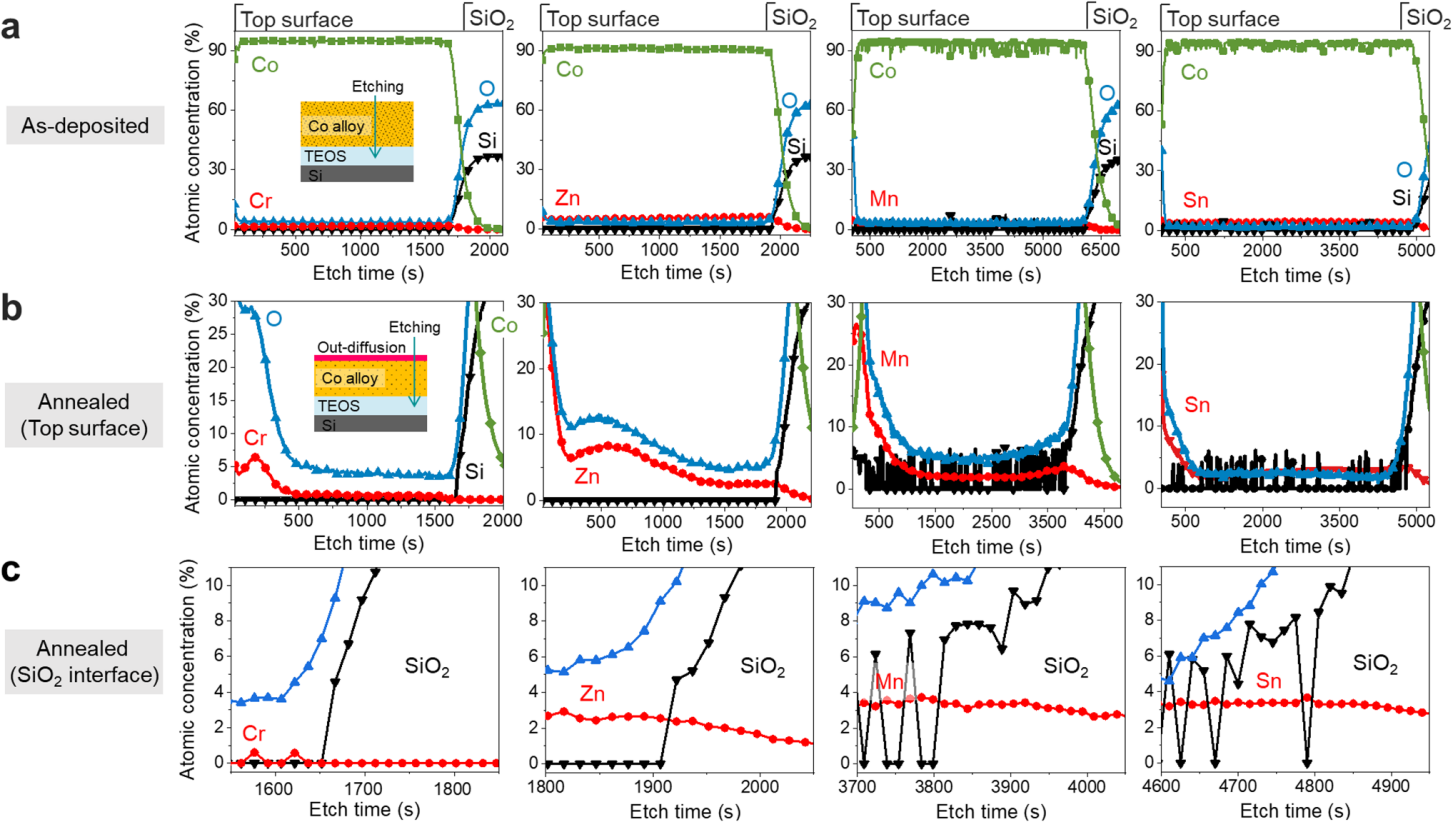
XPS depth profiles of Co alloy thin films; Co–Cr, Co–Zn, Co-Mn, and Co–Sn. (**a**) as-deposited thin films. (**b**) top surface profile after annealing at 450 °C. (**c**) alloy dopant profiles at the SiO_2_ interface after annealing.

Figure 2a shows X-ray photoelectron spectroscopy (XPS) depth profile results for thin films of Co–Cr, Co–Zn, Co–Mn, and Co-Sn alloys. A thin film was deposited to induce diffusion to the surface during annealing. Therefore, assuming the alloying element exhibits diffusion on the surface, the same result can be expected at the interface. Before annealing, each alloying element was uniformly doped. Since the alloy was deposited using the chip-on-target method, the doping concentrations of Cr, Zn, Mn, and Sn were confirmed to be 1.6 at%, 5.5 at%, 2.7 at%, and 3.9 at%, respectively. Chip-on-target deposition is an effective method for forming alloys with various doping concentrations. Figure 2b shows XPS profiles from the top surface to the SiO_2_ interface after heat treatment. Total XPS depth profiles of annealed samples at 450 °C was in Supplementary Information, Fig. S4. It was confirmed that all four alloying elements had moved to the top surface. The alloying elements diffused out of the Co matrix, suggesting that the design of the Co self-forming barrier was effective.

As shown in Table 1, Cr has a very high activity coefficient (68.572) and a high susceptibility toward oxidation, so the out-diffusion behavior of Co–Cr alloys occurred readily during the annealing process. On the other hand, since the activity coefficients of Zn and Mn are 0.997 and 0.463, respectively, their driving forces for surface diffusion are low. However, it can be expected that Zn and Mn would provide self-forming barriers because the driving forces for reaction with oxygen were high enough. Although Sn forms an IMC, it seems that the high driving forces for diffusion and oxidation would lead to self-forming barriers. When the alloying element has a higher activity coefficient and oxidation susceptibility, their influence on the self-formation of a barrier behavior seems to be dominant. The Co–Fe alloy did not diffuse to the surface. Energy dispersive X-ray spectroscopy (EDS) mapping (Fig. S5) indicated that Fe was still present in the Co matrix after annealing. The Co–Fe alloy does not diffuse on the surface because Co and Fe are representative ferromagnetic materials, and they tend to mix well with each other^28^.

Figure 2c shows the XPS profile for the SiO_2_ interface after annealing. The profiles of the alloying elements were different before and after annealing. Since it is deposited by sputtering, metal penetration may occur into the dielectric. Mn and Zn were still present in the SiO_2_ area after annealing. On the other hand, Cr did not remain in SiO_2_ after heat treatment and existed only at the interface. These results were also in good agreement with those from thermodynamics calculations. At the interface between Co and SiO_2_, the stable phases of Cr, Mn, and Zn were Cr_2_O_3_, MnSiO_3_ compound, and Zn_2_SiO_4_ respectively. Compared with the XPS results, Mn and Zn remained in the SiO_2_ region because they also reacted with Si to form silicates. On the other hand, Cr is an oxide former and does not react with Si, so it seems to have formed a clean interface with SiO_2_. From XPS analysis and the design rule, Cr exhibited the most suitable self-forming barrier behavior in Co interconnects. Cr and other alloying elements (Zn, Mn, Sn) also showed the production of self-forming barriers. In particular, Cr, which has a high activity coefficient, formed a clean interface and is expected to form chromium oxide. Considering that Fe exhibits unique magnetic properties like Co, the thermodynamic material design of this study itself is very effective.

**Figure 3:**
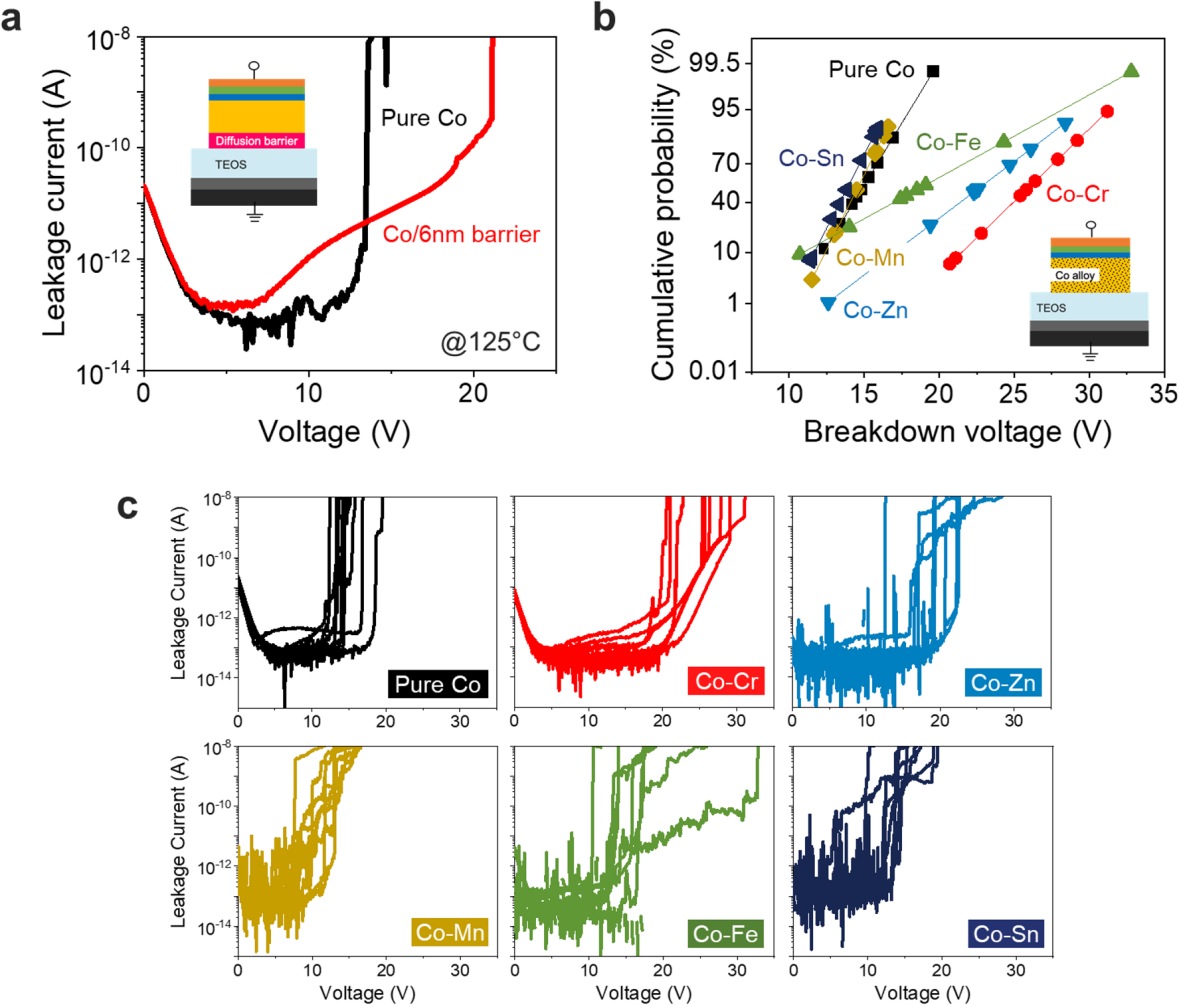
Voltage ramped dielectric breakdown (VRDB) analysis of pure Co and Co alloy MIS structures. (**a**) diffusion barrier effect; the leakage current shape is different for pure Co and Co/6 nm barriers. this is because the current conduction mechanism varies depending on the metal filament formation behavior. (**b**) cumulative distribution function (CDF) plot of breakdown voltage of pure Co and Co alloys. (**c**) I–V characteristics of pure Co and Co alloys after annealing at 450 °C. Electrical failure (breakdown voltage) was defined when leakage current increased over 10^–8^ A. Breakdown voltage values of the x-axis were converted to breakdown electric field values in Fig. S7.

Figure 3a shows the breakdown voltages measured for metal–insulator-semiconductor (MIS) structures to which a pure Co/6 nm diffusion barrier was applied. This 6 nm barrier consisted of a 3 nm TaN barrier and a 3 nm Ta liner, which are currently used in damascene interconnects. In this study, when the leakage current exceeds 10^–8^ A, the voltage was defined as the breakdown voltage (V_BD_). In the case of pure Co, abrupt dielectric breakdown occurred before 15 V, whereas in the case of the Co/6 nm barrier, dielectric breakdown occurred at 23 V. Early extrinsic breakdown by Co ions has been reported for pure Co since 2017, and the same result was confirmed in this study^16,17^. When a 6 nm barrier was formed, there was a difference in leakage current shape compared to that of Co/TEOS because current conduction was changed. In general, it has been reported that Schottky emission, Poole–Frenkel emission, and F-N tunneling current conduction occur when a Ta/TaN barrier is applied to Cu interconnects (the so-called “barrier effect”)^29,30^. When a Co alloy forms a barrier during annealing through interfacial diffusion and oxidation, leakage current conduction by the barrier effect will be different. Figure 3b shows the breakdown voltages of pure Co and Co alloy as a cumulative distribution function (CDF) graph. Compared with that for the pure Co sample, the breakdown voltages of Co-Cr, Co-Zn, and Co-Fe alloys were improved. The Co-Cr alloy showed the highest breakdown voltage and the smallest V_BD_ variation. The Co–Zn alloy and Co-Fe alloy showed excellent V_BD_ characteristics, but the variations were large. In the cases of Co-Sn and Co-Mn alloys, V_BD_ values lower than that of pure Co were confirmed. In general, high V_BD_ characteristics indicate high TDDB resistance, so the Co-Cr alloy showing a high V_BD_ is expected to significantly improve the TDDB lifetime^31^.

Figure 3c shows I–V results for pure Co and each Co alloy. In the cases of Co–Cr and Co–Zn, changes in current conduction were observed due to the barrier effect. In particular, the Co–Cr alloy showed excellent barrier quality. The current conduction mechanism for the Co–Cr alloy showed changes in Poole–Frenkel emission and F–N tunneling current conduction according to the E-field. In this regard, more accurate analyses of current conduction are in progress. In the case of the Co–Zn alloy, the current conduction behavior was not uniform, so it is expected that a conformal diffusion barrier was not formed at the interface. In the case of Co–Cr, breakdown voltages of up to 31.2 V were observed, suggesting that a Cr_2_O_3_ self-forming barrier with excellent diffusion barrier properties was formed. This result fits well with the thermodynamic material design. When Cr is applied to Co interconnects, it is expected that a Cr_2_O_3_ self-forming barrier with a high density and low diffusion coefficient will be generated through interfacial diffusion and bonding with oxygen. Since it has been proved experimentally to be a diffusion barrier that prevents metal ion penetration well, it can be an important barrier for Co interconnects. Since Co–Mn and Co–Sn alloys showed increases in leakage current from the 6 V region, there was no self-forming barrier effect. Co–Fe alloys showed higher V_BD_ values than pure Co but did not confirm the changes in leakage current shapes due to a barrier effect.

**Figure 4:**
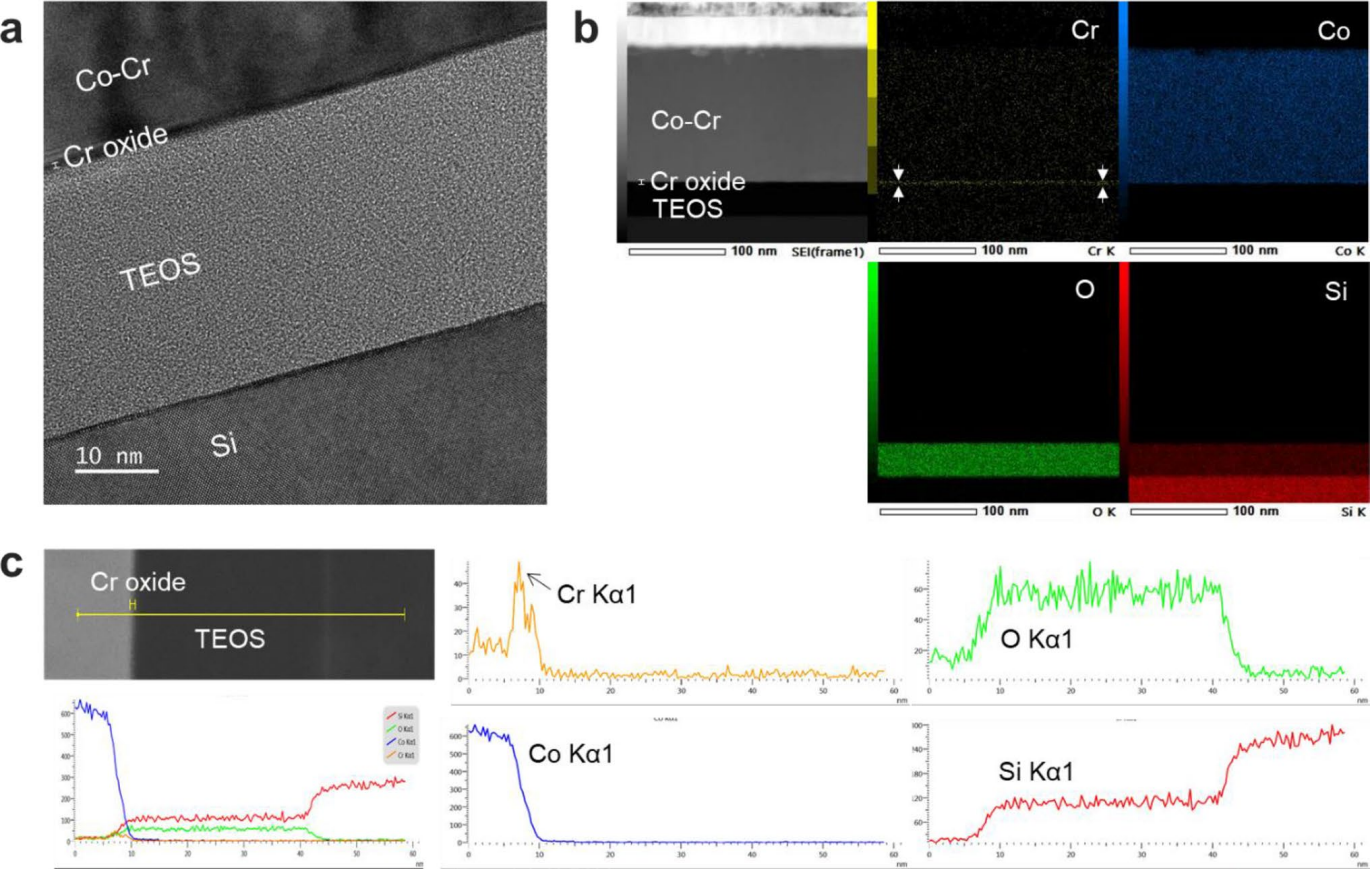
(**a**) High-resolution transmission electron microscopy (HR-TEM) images of Co-Cr MIS structure. (**b**) EDS mapping images; Co, Cr, O, and Si elements were analyzed at each interface. (**c**) EDS line scan.

Figure 4a shows an HR-TEM analysis of the interface of the Co–Cr alloy after annealing. A ~ 1.2 nm thick layer, which is expected to be a Cr_2_O_3_ layer, was confirmed at the TEOS interface. Cross-validation using various analytical instruments is in progress to confirm that the interfacial phase is Cr_2_O_3_. Considering the results of thermodynamic calculations and VRDB, it is expected that a Cr_2_O_3_ self-forming diffusion barrier was formed (Fig. S6) and enhanced the breakdown voltage. Since a very thin diffusion barrier layer is absolutely required for tens of nanometers of metal pitch, an ultrathin, highly reliable Cr_2_O_3_ self-forming barrier fully meets the barrier standards for Co interconnects. Figure 4b shows a TEM-EDS mapping image for the Co-Cr alloy exhibiting high V_BD_ characteristics. After annealing, Cr migrated from the Co matrix to the TEOS interface. Through EDS analysis, it was confirmed that Cr, which does not form an IMC phase and has a high activity coefficient, exhibited interfacial diffusion during the annealing process. Figure 4c shows the results from an analysis of the EDS line profile at the Co-Cr alloy interface with TEOS. After annealing, the Cr Kα1 peak significantly increased in intensity at the TEOS interface. Because the Co Kα1 peak decreased rapidly at the interface, it is clear that Cr diffused more effectively than Co.

Additionally, to understand the behavior of the Cr_2_O_3_ self-forming barrier at the interface, it is necessary to study the mechanism for Cr_2_O_3_ barrier formation and growth according as a function of annealing temperature, time, and the amount of Cr in the metal volume ratio at the interface. One of the reasons why Co interconnects with low resistance and relatively low processing challenges are not currently being utilized is the extrinsic failure caused by metal ions. Therefore, the design proposed herein for a self-forming diffusion barrier material for Co interconnects is very meaningful. Co-Cr alloy is the most suitable alloy material for a very narrow metal pitch because it generates a self-forming barrier with excellent diffusion barrier properties and ultrathin width. In conclusion, Co-Cr alloys could contribute to solving the extrinsic failure problems of Co interconnects.

## Conclusions

Self-forming barrier materials were designed to enhance the reliability of Co interconnects. By considering thermodynamic parameters such as oxidation formation energy, interfacial stable phases, intermetallic compound formation, solubility limits, and activity coefficients, it was confirmed that Co-Cr alloy is the most suitable self-forming barrier. The Co alloy design was in good agreement with the barrier property evaluations, which showed that it is an effective thermodynamic material design for self-forming diffusion barriers in Co interconnects. In the electrical evaluation, the Co-Cr alloy reacted with oxygen after interfacial diffusion and showed the best self-forming barrier behavior. It was proven through thermodynamic calculations and experiments that the Co-Cr alloy generated an ultrathin Cr_2_O_3_ self-forming barrier with a thickness of 1.2 nm during annealing. The Cr_2_O_3_ diffusion barrier formed at the dielectric interface had a very clean interface profile, and the breakdown voltage characteristics were improved by more than 200% compared to those of pure Co. Since Co-Cr alloy formed an interfacial diffusion barrier through Cr interfacial diffusion and oxidation, it will be sufficiently applicable to porous low-k dielectrics. When Cr_2_O_3_, which is known to retard metal cation emission due to its high density and low diffusion coefficient, is applied as a self-forming barrier to Co interconnects, it will prevent extrinsic failure and greatly improve interconnect reliability.

## Methods

A *p*-type silicon (100) wafer (resistivity: 1–10 Ω·cm) was used to fabricate the thin film sample and MIS device. Cleaning with a sulfuric acid-peroxide mixture (SPM) and dilute hydrofluoric acid (DHF) were performed to remove the native oxide. The cleaned substrates were thoroughly rinsed with deionized water (DIW) and dried using a wafer spin dryer. The SiO_2_ films were thermally grown on a Si wafer to a thickness of 100 nm by dry oxidation method for the thin film sample. In the case of the MIS device, a 30 nm thick TEOS dielectric (tetraethoxysilane, Soulbrain Co. Ltd.) was deposited via the CVD method. Thin films and MIS structures were fabricated using a DC magnetron sputtering deposition system (ULTECH Co.,) with the chip-on-target method. The chip-on-target method is the same as other common sputtering systems except that small chips are located on the main target. An actual picture and schematic of the chip-on-target method are presented in Fig. S2. The reason for using the chip-on-target method is that alloy properties can be quickly verified without creating a target for each composition. After that, Ta passivation was used as a metal capping layer to increase the driving force for reaction with oxygen at the SiO_2_ interface^23^. The Al bottom electrode was deposited to a thickness of 500 nm. An 80 nm Au top electrode and a 20 nm Ti adhesion layer were used after the Ta process. A sample annealing process (450 °C, 2 h) was performed. In the case of thin film samples, heat treatment was performed in a vacuum chamber to induce surface diffusion. Next, the MIS device was subjected to wafer-level annealing in an N_2_ atmosphere.

Binary and ternary phase systems calculated with a thermochemical database program (Factsage™ 7.3 and 8 software) were used to determine solubility, intermetallic compound (IMC) formation, activity coefficients, and interfacial stable phases of the Co-X system^32,33^ (detailed in Supplementary Information).

The element composition ratio and depth profile of the thin film were measured using a photoelectron spectrometer (NEXSA, Thermo Fisher Scientific) located at the Jinju Center of the Korea Institute of Ceramic Engineering and Technology (KICET). An etching process was first performed for 30 s to remove surface contamination. The sputter energy was fixed at 2 kV which can be measured as a 0.4 nm/sec sputter rate for Ta_2_O_5_ during etching for the depth profile of Co alloys. Measurement data were corrected with the 284.6 eV C1s peak to remove hydrocarbon noise. The I–V characteristics (VRDB) of the fabricated devices were measured using a probe station (Modusystems, Inc., and MSTECH) and a semiconductor parameter analyzer (Keithley 4200A-SCS). For the I–V curve, the voltage was swept from 0 to −80 V in steps of −100 mV at 125 °C. Analytical TEM (JEM-F200(TFEG), 2100F, and Tecnai F20) and an energy dispersive X-ray spectrometer (Oxford EDS) were used to obtain the HRTEM images, line EDS, and EDS mapping.

## Supplementary Information

Supplementary Information.

## Data Availability

The datasets used and analyzed during the current study available from the corresponding author on reasonable request.
