# Supplementary material for: Robust Co alloy design for Co interconnects using a self-forming barrier layer
Source: Sci Rep. 2022 Jul 19;12:12291. doi: 10.1038/s41598-022-16288-y (PMC9296516; doi:10.1038/s41598-022-16288-y)
Supplement: Supplementary file 1 — Supplementary Information. [file 41598_2022_16288_MOESM1_ESM.docx]

Supplementary Information

**Robust Co Alloy Design for Co Interconnects using a Self-Forming Barrier Layer**

Cheol Kim,^1,⸸^ Geosan Kang,^1,⸸^ Youngran Jung,^1^ Ji-Yong Kim,^1^ Gi-Baek Lee,^1^ Deokgi Hong,^1^ Yoongu Lee,^1^

Soon-Gyu Hwang,^1^ In-Ho Jung,^1^ and Young-Chang Joo^1,2*^

*^1^Department of Materials Science & Engineering, Seoul National University, Seoul, 08826, Republic of Korea
^2^Research Institute of Advanced Materials (RIAM), Seoul National University, Seoul, 08826, Republic of Korea*
^*^ycjoo@snu.ac.kr
(⸸ these authors equally contributed.)

***Corresponding authors:**

Young-Chang Joo*

E-mail: ycjoo@snu.ac.kr Tel: +82-2-880-8986, Fax: +82-2-883-8197

*Department of Materials Science & Engineering, Seoul National University, Seoul, 08826, Republic of Korea*

**Thermodynamic calculation.**

The Co-metal (X) system's oxide formation energy, intermetallic compound (IMC) production, solubility, activity coefficients, and interfacial stable phases were determined using binary and ternary phase systems computed with a thermochemical database program (Factsage^TM^ 7.3 and 8 software). In this software, appropriate pure substance databases and solution databases in the program were chosen for thermodynamic model equations consisting of temperature and compositions such as mole fraction or partial pressures.^1^  Through these variables of databases, the thermochemical calculation processes were conducted in order to minimize the Gibbs free energy (G) in binary or ternary systems by following the equations.^1, 2^

$$\boldsymbol{G=}\sum_{\boldsymbol{Ideal gas}} \boldsymbol{n}_{\boldsymbol{i}}\left( \boldsymbol{g}_{\boldsymbol{i}}^{\boldsymbol{o}}\boldsymbol{+RTlog}\boldsymbol{P}_{\boldsymbol{i}} \right)\mathbf{+}\sum_{\boldsymbol{Pure phase}} \boldsymbol{n}_{\boldsymbol{i}}\boldsymbol{g}_{\boldsymbol{i}}^{\boldsymbol{o}}\mathbf{+}\sum_{\boldsymbol{Solution 1}} \boldsymbol{n}_{\boldsymbol{i}}\left( \boldsymbol{g}_{\boldsymbol{i}}^{\boldsymbol{o}}\boldsymbol{+RTlog}\boldsymbol{\gamma}_{\boldsymbol{i}}\boldsymbol{X}_{\boldsymbol{i}} \right)\boldsymbol{+}\sum_{\boldsymbol{Solution 2}} \boldsymbol{n}_{\boldsymbol{i}}\left( \boldsymbol{g}_{\boldsymbol{i}}^{\boldsymbol{o}}\boldsymbol{+RTlog}\boldsymbol{\gamma}_{\boldsymbol{i}}\boldsymbol{X}_{\boldsymbol{i}} \right)\boldsymbol{+ \cdots}$$

($\boldsymbol{n}_{\boldsymbol{i}}\boldsymbol{=}\mathbf{moles}\boldsymbol{,}\boldsymbol{g}_{\boldsymbol{i}}^{\boldsymbol{o}}\boldsymbol{=}\mathbf{standard molar Gibbs energy}\boldsymbol{,}\boldsymbol{P}_{\boldsymbol{i}}\boldsymbol{=}\mathbf{partial pressure,}$

$\boldsymbol{\gamma}_{\boldsymbol{i}}\boldsymbol{=}\mathbf{activity coefficient,} \boldsymbol{X}_{\boldsymbol{i}}\boldsymbol{=}\mathbf{mole fraction}$)

We used the pure substances database (FactPS), the oxide compound database (FToxid), the FScopp was selected first, and the other solution databases were selected for the elements that have a limit in the binary phase of the FScopp database. We additionally selected the FTlite, FSstel, SGTE, SGnobl, and SpMCBN databases.

The Gibbs free energy of each compound was calculated at 450 °C, 1 atm for the Co-X alloy binary and ternary systems. The thermodynamic calculations were conducted under the assumption independent of time until the optimization of Gibbs energy. For the oxide formation energy, the Gibbs free energies of each metal’s oxidation reaction were calculated according to the temperature with the balance of the reaction formula with O_2_ fixed by 1 mole and compared. To figure out which Co-X alloy could intermetallic compound at a given temperature, phase diagrams of the Co-X binary system were analyzed using database documentation in Factsage^TM^ 8. The possibility of IMC formation of each metal element was determined according to whether at least one of the compounds excluding the solid solution was present. Some elements can be solute in the phase without intermetallic compounds until specific points when alloys amounts are small, but if the temperature is getting low, it could change to intermetallic compounds. These metals cannot determine the correct concentration as an alloy, so these elements were excluded for metal candidates.

For calculating the solubility, activity coefficient, and interfacial phases, a total of 1 mole of Co and alloy metal with 1 mole of SiO_2_ were reacted for phase equilibrium at the ternary system. The maximum solubility of each metal in Co was calculated as the point of mole fraction of metal when their solid solution phase (FCC) changed to another solid solution phase (HCP) appeared. Interfacial stable phases were calculated within the solubility limit of the metal in ternary systems (Metal-SiO_2_-Co). Zn, Fe, and Mn can make interfacial stable compounds as silicate, and Cr can form Cr Oxide. In Ni’s case, the reaction product between Ni and SiO_2_ was formed as silicate in a binary system but there was no product in ternary systems. The activity coefficients of each metal were calculated from the activity value of their solid solution phase and solubility limits by the following equation, Activity a = γN_X_ (γ: activity coefficient, N_X_: solubility limits).

Table S1. Calculation of interfacial stable phase at annealing temperature (450 °C) using Factsage^TM^: The thermodynamic stable phase of each element was calculated in binary system (M-SiO_2_) and ternary system (M-SiO_2_-Co).

| Element | Binary system @450 ℃  (M-SiO_2_) | Ternary system @450 ℃  (M-SiO_2_-Co) |
| --- | --- | --- |
| Ag | X | - |
| Au | X | - |
| Cr | Cr_2_O_3_ | Cr_2_O_3_ |
| Cu | X | - |
| Fe | Fe_2_SiO_4_ | Fe-Co-Si compound |
| Ge | X | - |
| Mn | MnSiO_3_ | MnSiO_3_ + CoSiO_3_ compound |
| Ni | Ni_2_SiO_4_ | X |
| Pd | X | - |
| Pt | X | - |
| Sn | SnO_2_ | SnO_2_ |
| Ru | X | - |
| Zn | Zn_2_SiO_4_ | Zn_2_SiO_4_ |

Table S2. Activity coefficient of each dopant in the Co matrix at 450 °C: dopants in the Co matrix are less stable when the activity coefficient is greater than 1. Activity coefficients were calculated using Factsage^TM^ software.

| Element | Activity coefficient (γ) |
| --- | --- |
| Al^1)^ | 3.330E-06 |
| As^1)^ | 3.023 |
| Be^1)^ | 3.217 |
| Cd^1)^ | 1.094E+05 |
| Cr^*,1)^ | 68.572 |
| Cu^*,1)^ | 1.428E+03 |
| Fe^1)^ | 0.263 |
| Ga^2)^ | 8.150E-04 |
| Ge^1)^ | 3.137 |
| Mn^1)^ | 0.463 |
| Mo^*,3)^ | 2.075 |
| Ni^*,1)^ | 0.999 |
| Pd^1)^ | 1.29E-08 |
| Pt^*,1)^ | 0.999 |
| Sn^*,1)^ | 2.653 |
| Ta^3)^ | 3.89E-07 |
| Ti^1)^ | 3.75E-06 |
| V^2)^ | 4.898E-03 |
| Zn^*,1)^ | 0.997 |

Material database: 1) FScopp, 2) SGTE, 3) FTlite, 4) FTstel, 5) spMCBN, 6) SGnobl

Table S3. Solubility and intermetallic compound (IMC) information for 39 elements. Values were calculated using Factsage^TM^ software.

| Element | Solubility  (at% @450°C) | Intermetallic  compound formation  (Y/N) | Element | Solubility  (at% @450°C) | Intermetallic  compound formation  (Y/N) |  |
| --- | --- | --- | --- | --- | --- | --- |
| Ag^1)^ | No | N | Mo^4)^ | 0.04 | Y | |
| Al^1)^ | 1.58 | Y | Na^3)^ | 4.96E-07 | liquid | |
| As^1)^ | 3.11 | N | Nb^1)^ | 0.108 | Y | |
| Au^2)^ | 0.14 | N | Ni^1)^ | 100 | N | |
| B^3)^ | No | Y | Pd^1)^ | 4.02 | N | |
| Be^1)^ | 0.19 | N | Pt^1)^ | 100 | N | |
| Bi^1)^ | No | liquid | Re^5)^ | 0.417 | N | |
| Ca^1)^ | No | N | Rh^4)^ | 3.427 | N | |
| Cd^1)^ | 6.79E-04 | liquid | Ru^6)^ | ~0.3 | N | |
| Cr^1)^ | 0.305 | N | Sb^2)^ | No | Y | |
| Cu^1)^ | 0.07 | N | Sc^5)^ | 0.2 | Y | |
| Fe^1)^ | 10.11 | N | Sn^1)^ | 2.12 | Y | |
| Ga^2)^ | 10.834 | Y | Ta^4)^ | 1.24 | Y | |
| Ge^1)^ | 1.17 | N | Ti^1)^ | 0.635 | Y | |
| Hf^3)^ | No | Y | V^2)^ | 1.7 | Y | |
| In^1)^ | No | Y | W^1)^ | No | Y | |
| K^4)^ | X | liquid | Y^2)^ | No | Y | |
| Li^4)^ | No | liquid | Zn^1)^ | 4.88 | N | |
| Mg^1)^ | No | Y | Zr^1)^ | No | Y | |
| Mn^1)^ | 8.11 | N |  |  |  | |

Material database: 1) FScopp, 2) SGTE, 3) FTlite, 4) FTstel, 5) spMCBN, 6) SGnobl


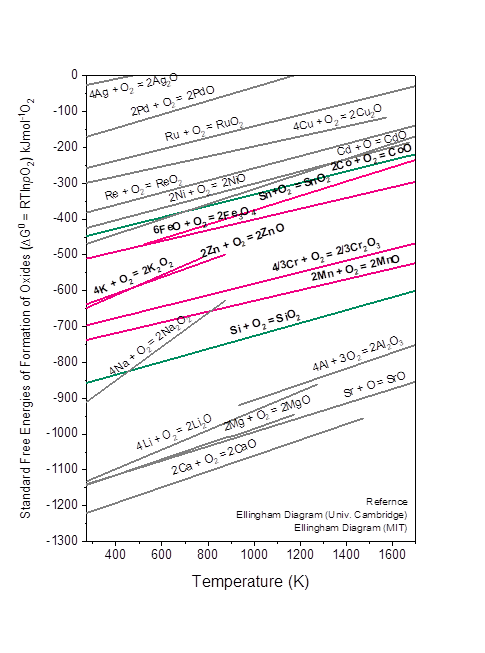


Figure S1. Ellingham diagram of elements. For appropriate self-forming barrier in the Co alloy system, the oxidation tendency of alloy metal should be higher than Cobalt and lower than SiO_2_ to react with Oxygen and avoid excess Si. These elements were marked in the graph as pink color.


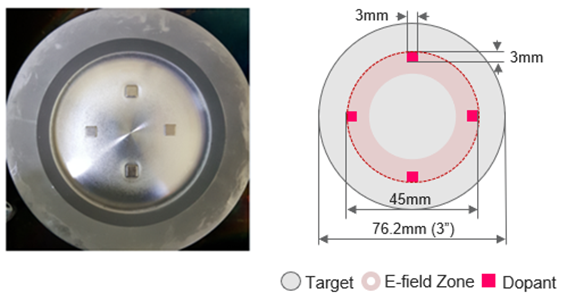


Figure S2. The picture and schematic figure which described chip-on-target deposition method. Small chips are located on the target to control the content of alloying dopants.


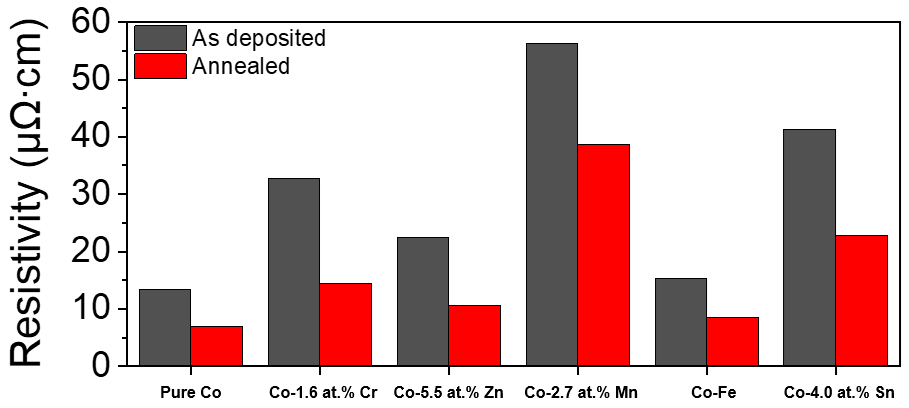


Figure S3. Electrical resistivity of the studied alloy films. All films were annealed at 450 °C for 2 hours.


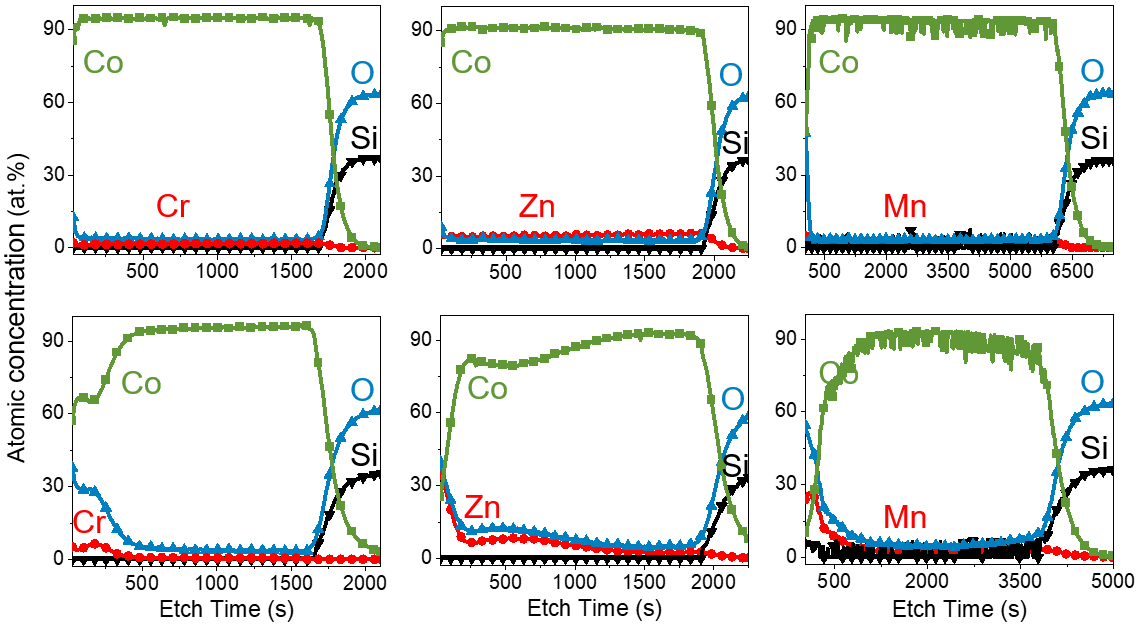


Figure S4. Total XPS depth profile of the as-deposited (top) and annealed sample (bottom). All films were annealed at 450 °C for 2 hours.


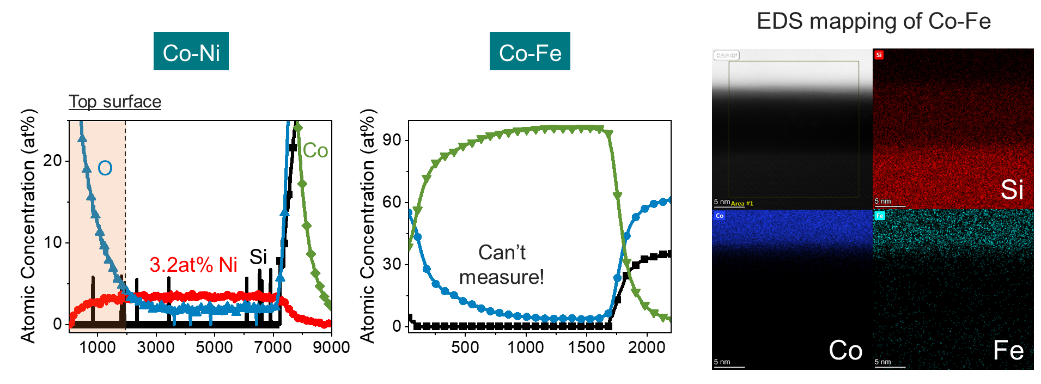


Figure S5. XPS depth profiles of Co-Fe and energy dispersive X-ray spectroscopy (EDS) mapping of Co-Fe alloy; In the case of the Co-Fe alloy, it was difficult to distinguish the two elements by XPS analysis because the binding energies of Co and Fe overlap.


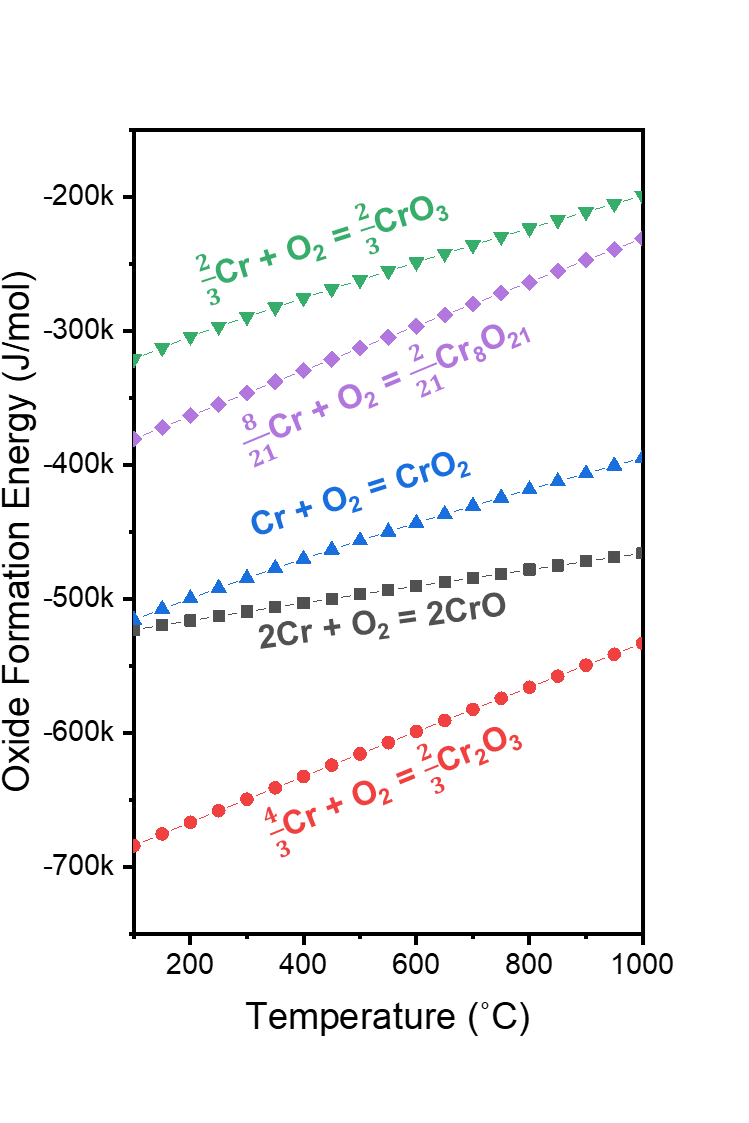


Figure S6. Gibbs free energy comparison of several Cr oxides formation as a function of temperature. Thermodynamic relative stability could be inferred that Cr_2_O_3_ phase was the most stable among Cr oxide from these values.


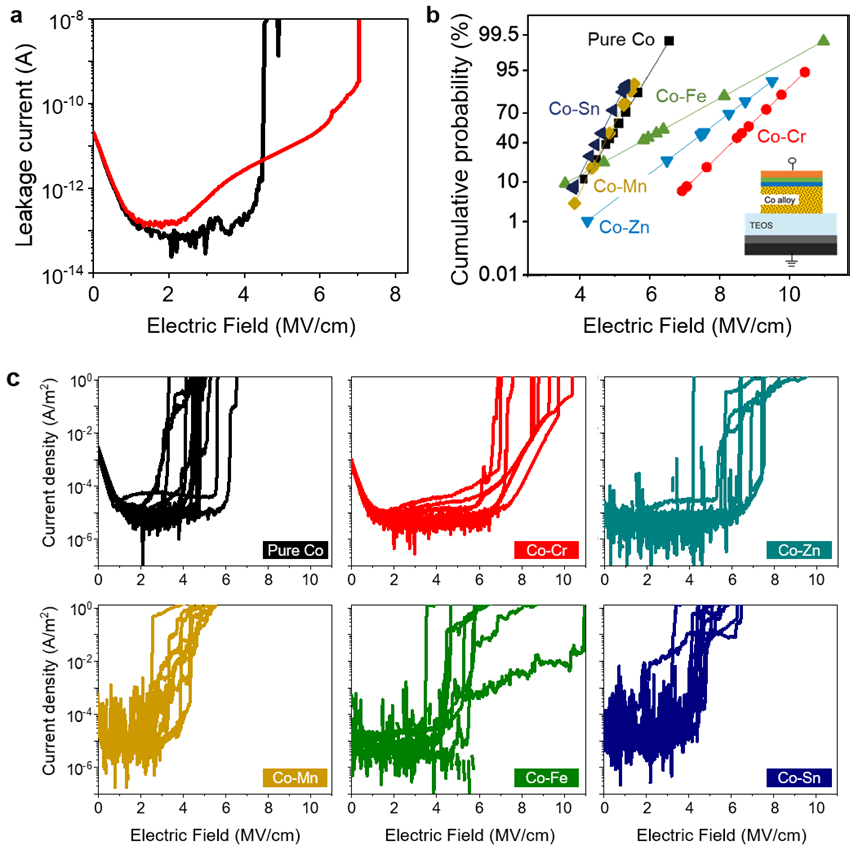


Figure S7. Voltage ramp dielectric breakdown (VRDB) analysis of pure Co and Co alloy MIS structures plotted as a function of the electric field.

**References**

1. Factsage^TM^ 8.0 software, Equisage, <https://www.factsage.com>.
2. Gaskell, D.R., & Laughlin, D.E. Introduction to the Thermodynamics of Materials (6th ed.). CRC Press. (2017)
